# Investigating the contribution of rare non-coding variants in *BRCA1*, *BRCA2* and *PALB2* to hereditary breast cancer

**DOI:** 10.1038/s41523-026-00942-z

**Published:** 2026-04-04

**Authors:** Qihong Zhao, Na Li, Evanny Marinovic, Simone McInerny, Maia Zethoven, Lisa Devereux, Daffodil M. Canson, Amanda B. Spurdle, Rodney J. Scott, Paul A. James, Ian G. Campbell

**Affiliations:** 1 Peter MacCallum Cancer Centre, Melbourne, VIC Australia; 2 Sir Peter MacCallum Department of Oncology, The University of Melbourne, Melbourne, VIC Australia; 3 Parkville Familial Cancer Centre, Peter MacCallum Cancer Centre and Royal Melbourne Hospital, Melbourne, VIC Australia; 4 QIMR Berghofer, Herston, QLD Australia; 5 Discipline of Medical Genetics and Centre for Information-Based Medicine, The University of Newcastle and Hunter Medical Research Institute, Newcastle, NSW Australia; 6 Division of Molecular Medicine, Pathology North, Newcastle, NSW Australia

**Keywords:** Cancer, Genetics, Molecular biology, Oncology

## Abstract

Pathogenic coding variants in *BRCA1*, *BRCA2* and *PALB2* confer hereditary breast/ovarian cancer risk, yet these regions comprise less than 10% of the genomic footprint of these genes, leaving most sequence unexplored. We investigated the contribution of non-coding variation to hereditary breast cancer by analyzing intronic variants and 5′ upstream regions of *BRCA1*, *BRCA2* and *PALB2* in the BEACCON case–control study of over 11,000 participants. Full-gene sequencing showed that 46.3% of cases carried at least one rare non-coding variant. This was associated with a modest increase in breast cancer risk (OR = 1.2, *p* < 0.0001), most likely reflecting the presence of a small proportion of pathogenic variants within a larger background of predominantly neutral variation. Stronger enrichment was observed for triple-negative disease, particularly for *BRCA1* (OR = 1.5, *p* = 0.0001). Tumor sequencing of 42 high-priority variants identified 11 (26.2%) with wild-type allele loss and high homologous recombination deficiency. Functional CRISPR/Cas9 knock-in assays in MCF10A cells confirmed that two deep intronic variants created aberrant splice sites, disrupted splicing and impacted transcript expression.

## Introduction

Approximately 5-10% of breast cancer (BC) cases are hereditary with germline pathogenic variants in *BRCA1*, *BRCA2* and *PALB2* accounting for 20–25% of these families^1^. Other hereditary breast cancer (HBC) genes, such as *ATM* and *CHEK2,* collectively explain less than 5% of HBC cases^2^. Consequently, many families investigated through genetic testing for HBC do not have a pathogenic variant identified in any known HBC gene. Despite some advances in identifying additional HBC genes, more than 70% of HBC cases remain unexplained by known susceptibility genes^3^ which compromises risk management for the index case and precludes cascade testing in blood relatives.

The identification of new HBC genes has advanced little since the discovery of *BRCA1* and *BRCA2* more than 3 decades ago, with *PALB2* being an additional gene since shown to confer moderate-to-high penetrance risk. The results of whole exome sequencing (WES) studies of HBC cohorts indicate that the remaining genes associated with HBC are likely to either account for a very small proportion of families or have only modest risk effects, making them extremely difficult to identify^4,5^. To increase the power to discover rare novel HBC genes the BEACCON study sequenced 1303 candidate genes in 5770 BC patients from families exhibiting strong hereditary characteristics but with no identified pathogenic variants in known HBC genes, and 5741 matched cancer-free controls^6^. Utilizing a case–control statistical framework, the BEACCON study identified multiple potential HBC susceptibility genes, but even the top candidate genes remained exceptionally rare in this large cohort. For instance, while the odds ratio (OR) for the candidate HBC gene *BLM* was 2.49, indicating moderate-to-low association with BC risk, loss-of-function (LoF) variants in *BLM* were found in only 20 cases (0.35%) compared to 8 (0.14%) in controls. In this study, 67% of patients did not harbor a LoF variant in any of the tested genes, which included all genes with a function related to DNA repair or genome maintenance^6^. Another possibility raised by these findings is that many HBC families might harbor pathogenic abnormalities in already known HBC genes, but because the variants are located in non-coding regions not routinely interrogated in either clinical practice or most research studies, these have remained unidentified. Indeed, statistical modelling using a maximum likelihood estimation approach has been performed on extended sequence data for *BRCA1*, *BRCA2* and *PALB2* from BEACCON participants, and provided significant evidence of pathogenic variants among rare non-coding variants, in particularly in deeper intronic variants, with the estimated proportion of pathogenic variants ranging from 6 to 13%^7^.

Further, several small-scale studies have identified rare examples of deep intronic likely pathogenic variants in *BRCA1*^8^*, PALB2* and *ATM*^9^. Analysis of somatic drivers has also shown that non-coding variants can affect gene expression and splicing, as well as additional regulatory mechanisms such as altered RNA stability^10^ and promoter methylation, as reported for a *BRCA1* 5′UTR variant^11^.

Together, these studies underscore the importance of escalating the search for the missing heritability of BC in non-coding regions of established HBC genes.

Using existing germline *BRCA1*, *BRCA2* and *PALB2* extended gene sequence data from the BEACCON study of 5770 cases and 5741 controls^6^, this study explored the extent to which individual non-coding variants might explain the missing BC heritability. For BC-affected women who were found to harbor potentially pathogenic non-coding variants, tumor sequencing was performed to assess genomic features expected for carriers of pathogenic variants in these genes, and top candidate variants were introduced into a cell model for further characterization.

## Results

### Identification of rare non‑coding variants in *BRCA1*, *BRCA2* and *PALB2*

**Fig. 1:**
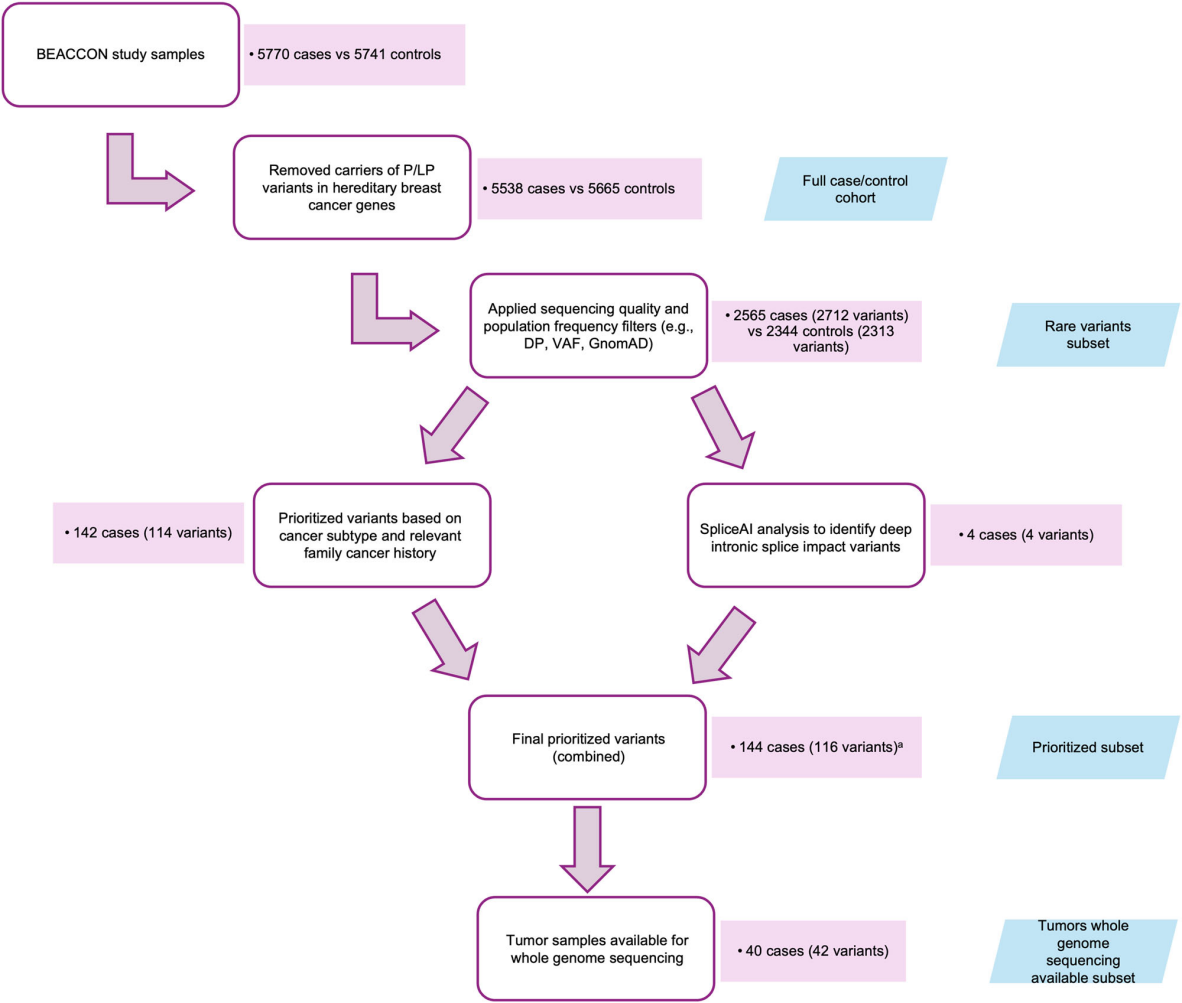
Flowchart illustrating the filtering, ranking, prioritization and curation steps used on the processed BEACCON study *BRCA1*, *BRCA2* and *PALB2* non-coding variants. Steps performed in the post-sequencing pipeline (i.e. alignment of FASTQ reads, variant calling and annotation) are not displayed. P/LP pathogenic/ likely pathogenic, DP allele sequencing depth, VAF Variant allele fraction. ^a^Two variants (two cases) overlap between the SpliceAI-identified set and relevant family cancer history set; they are counted once in the Final prioritized variants (combined) total.

The BEACCON study cohort was used to obtain sequencing data for the entire coding, non-coding and promoter regions of *BRCA1*, *BRCA2* and *PALB2*^6^. As reported previously, no significant coverage bias was observed between cases and controls at the individual gene level. As part of the BEACCON study analysis 232 cases and 275 controls were identified with a pathogenic or likely pathogenic variant in another HBC gene, and these subjects were excluded from the non-coding variant analysis, leaving 5538 cases and 5665 controls (Full case/control cohort). Population frequency filters were applied to remove common variants (population frequency > 0.0005 in the 1000 Genomes Project, ESP, or gnomAD) as these variants are unlikely to have effect sizes comparable to those of known pathogenic variants in *BRCA1*, *BRCA2*, or *PALB2*. In total, 4287 unique rare non-coding variants were identified across the cases and controls. Overall, 46.3% of cases (2565 rare variants carriers; termed the rare variants case subset) and 41.4% of controls (2344 rare variants carriers; termed the rare variants control subset) harbored at least 1 rare non-coding variant (OR = 1.22, *p* < 0.0001) in *BRCA1*, *BRCA2* or *PALB2*. Repeated internal cross-validation analyzes yielded consistently elevated OR across all randomly generated subsets (median OR = 1.32, range = 1.24–1.41), with all subsets showing OR > 1 and statistical significance at *P* < 0.05 (Table S7), indicating no evidence for the contribution of confounding factors to the overall results. As summarized in Table 1, for each gene examined, there was a small but significant excess of rare variants in the cases versus the controls. Of the case “carriers”, the majority harbored only one rare variant in any of three genes (70.3% of the rare variants case subset) with 21.0% carrying two variants and 8.7% carrying three or more variants. The complete list of these variants, together with details of the corresponding cases and controls, is provided in Table S3. An overview of the filtering, ranking, prioritisation and curation strategy applied to *BRCA1*, *BRCA2* and *PALB2* non-coding variants in the BEACCON study is shown in Fig. 1.

**Table 1:** Frequency of rare non-coding variants in *BRCA1*, *BRCA2*, and *PALB2* among cases and controls from the BEACCON study

Gene	Case subjects (*n* = 5538)		Control subjects (*n* = 5665)		OR (95% CI, *P*-value^b^)
	*N*^o^ of unique variants^a^	*N*^o^ of variant carriers (%)	*N*^0^ of unique variants	*N*^0^ of variant carriers (%)	
*BRCA1*	1163	1298 (23.44)	1007	1147 (20.25)	1.2 (1.1–1.3, <0.0001)
*BRCA2*	1196	1331 (24.03)	1011	1235 (21.80)	1.1 (1.0–1.2, 0.0050)
*PALB2*	353	441 (7.96)	295	364 (6.43)	1.3 (1.1–1.5, 0.0017)

^a^Number of non-coding variants after applying sequencing quality filters and removing variants reported in gnomAD at a frequency >0.0005. Additionally, variants with Genetic Ancestry allele frequency (GA-AF) greater than 0.01 in any ethnicity group were excluded.

^b^ORs, 95% confidence intervals (CIs), and Fisher’s exact p-values were calculated based on the number of carriers.

Because ER status and triple-negative breast cancers (TNBC) are strongly associated with *BRCA1* and *PALB2* pathogenic variants, and weakly with *BRCA2*, the frequency of non-coding variants was assessed among 2517 cases from the ViP study for which ER expression status was available (Table 2). Compared to the overall cases shown in Table 1, cases with known ER, PR and HER2 status showed higher frequencies of non-coding variants for each gene. Consistent with at least some of these variants being pathogenic, TNBC cases all showed higher frequencies of rare non-coding variants with the enrichment being more pronounced for *BRCA1* (27.5% of individuals with TNBC had a *BRCA1* variant versus 23.4% for any pathology).

**Table 2:** Frequency of rare non-coding variants in *BRCA1*, *BRCA2*, and *PALB2* among ER status known/TNBC cases and controls from the BEACCON study

Gene	Cases ER status known (*n* = 2517)		OR (95% CI, *p*-value)^b^	Cases with TNBC (*n* = 550)		OR (95% CI, *p*-value)^c^
	*N*^o^ unique variants^a^	*N*^o^ variant carriers (%)		*N*^o^ unique variants	*N*^o^ variant carriers (%)	
*BRCA1*	659	644 (25.59)	1.4 (1.2–1.5, <0.0001)	186	151 (27.45)	1.5 (1.2–1.8, 0.0001)
*BRCA2*	670	657 (26.10)	1.3 (1.1–1.4, <0.0001)	156	145 (26.36)	1.3 (1.1–1.6, 0.0156)
*PALB2*	201	234 (9.30)	1.5 (1.3–1.8, <0.0001)	55	53 (9.64)	1.6 (1.1–2.1, 0.0056)

^a^Number of non-coding variants after applying sequencing quality filters and removing variants reported in gnomAD at a frequency >0.0005. Additionally, variants with Genetic Ancestry allele frequency (GA-AF) greater than 0.01 in any ethnicity group were excluded.

^b^ORs, 95% confidence intervals (CIs), and Fisher’s exact p-values were calculated based on the number of carriers in the ER status known group and overall BEACCON control group given in Table 1.

^c^ORs, 95% confidence intervals (CIs), and Fisher’s exact p-values were calculated based on the number of carriers in the ER/PR/HER2 status known group and overall BEACCON control group given in Table 1.

### Identification of intronic splicing defects

The full list of rare variants identified in the cases and controls (4287) were analyzed for variants predicted to alter splicing using the SpliceAI algorithm. Using the SpliceAI recommended prediction score of ≥ 0.5 to achieve higher specificity, 4 variants were identified (all in cases) (Table S4) as having a high likelihood of causing splicing defects. For each gene there were more predicted splice-altering variants in cases versus the controls although none of these differences were statistically significant.

Given that several research groups have suggested lowering the SpliceAI threshold to improve sensitivity^9,12^, variants with scores of ≥ 0.2 were considered^13^. The lower score threshold identified 19 variants (15 in the cases and 4 in the controls, Table 3). In *PALB2*, two distinct predicted splice-altering variants (*PALB2* c.3114-239 A > T and c.3113+5 G > C) were identified across five cases; no splice-altering variants were observed in controls (OR = 11.3, *P* = 0.10). Details of the specific SpliceAI predicted variants are provided in Table S4. Overall, predicted splice-altering variants constituted only a small fraction of the rare non-coding variant set (15 unique variants) and affected 19 of 5538 cases (0.3% of full case cohort). Considering that using a SpliceAI threshold of 0.2 may introduce false-positive predictions, a threshold of 0.5 was retained for the final prioritization.

**Table 3:** Summary of predicted splice-altering variants in *BRCA1*, *BRCA2*, and *PALB2* identified by SpliceAI

Gene	Cases (*n* = 5538)		Controls (*n* = 5665)		OR (95% CI, *p* value)^b^
	*N*^o^ of variants^a^	*N*^o^ of carriers	*N*^o^ of variants	*N*^o^ of carriers	
*BRCA1*	9	10	2	2	5.1 (1.1–23.4, *p* = 0.0350)
*BRCA2*	4	4	2	2	2.1 (0.4–11.2, *p* = 0.4084)
*PALB2*	2	5	0	0	11.3 (0.6–203.6, *p* = 0.1013)
*Total*	15	19	4	4	4.9 (1.7–14.3, *p* = 0.0040)

^a^The full list of rare variants were analyzed using SpliceAI with delta scores of ≥ 0.2, after excluding variants classified as Benign in ClinVar and those with in gnomAD and with SpliceAI scores < 0.2 in gnomAD (v4.1.0).

^b^ORs and *p* values are calculated for comparison of the number of carriers of SpliceAI predicted variants in cases versus controls.

### Prioritization of case carriers for tumor sequencing

**Fig. 2:**
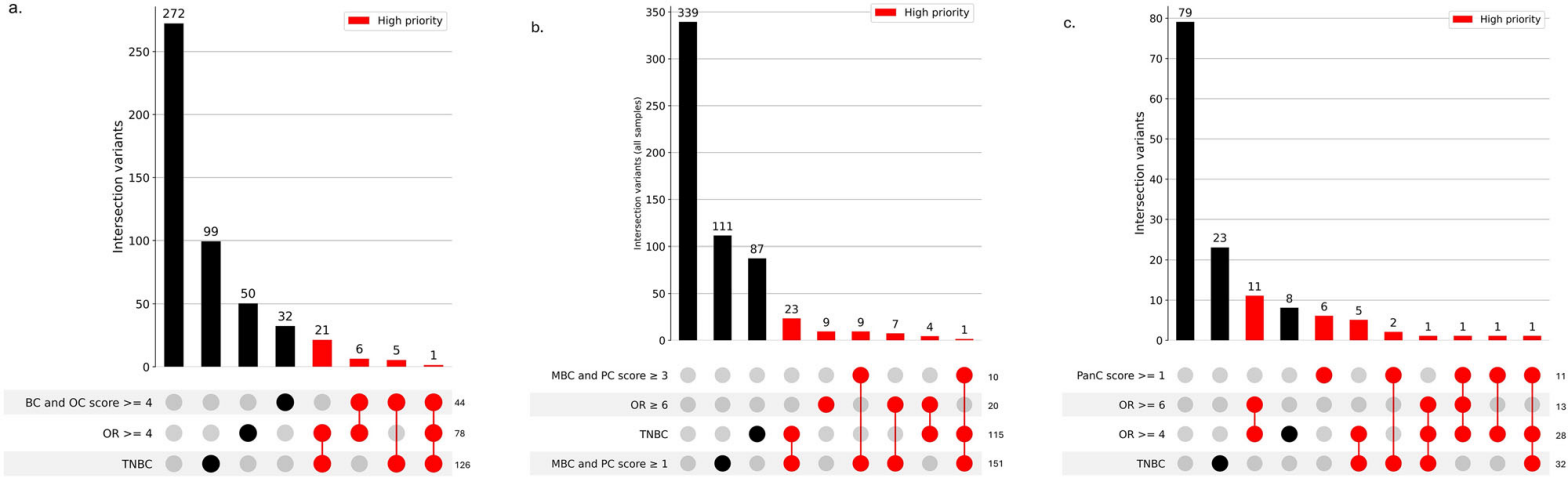
UpSet plots illustrating the intersection of priority variant selection criteria for *BRCA1*, *BRCA2*, and *PALB2.* Each plot shows the number of variants meeting combinations of high-priority criteria. Red bars highlight intersections that contain high-priority variants. All cancer types listed (e.g., BC, OC, TNBC, MBC, PC, PanC) refer to diagnoses in the personal or relatives within two degrees of relation (family history). Selection criteria include: **a** UpSet plot for *BRCA1*. BC breast cancer, OC ovarian cancer, TNBC triple-negative breast cancer, OR odds ratio. **b** UpSet plot for *BRCA2*. MBC: male breast cancer, PC prostate cancer. **c** UpSet plot for *PALB2*. PanC: pancreatic cancer. Bar heights indicate the number of variants fulfilling each intersection of criteria. Dots below the bar plot represent which criteria are included in each intersection.

In addition to identifying potential pathogenic intronic splice-altering variants, other variants were prioritized according to tumor characteristics and family history commonly associated with known pathogenic variants in *BRCA1*, *BRCA2* and *PALB2* as summarized in the upset plots (Fig. 2).

As summarized in Table 4, this filtering identified a total of 114 variants among 142 cases carriers with a rare variant in *BRCA1*, *BRCA2*, or *PALB2*, with all of these specific variants being very rare among the controls. These variants demonstrated increased odds ratios and were found in individuals with personal or familial cancer histories highly consistent with the associated gene-specific phenotypes. Two of the prioritized variants were also identified as likely splice altering variants (*BRCA1* c.5194-1112 C > T and *PALB2* c.3114-239 A > T).

**Table 4:** Number of rare non-coding variants in *BRCA1*, *BRCA2* and *PALB2* after filtering based on tumor characteristics, case personal and family cancer history and ORs

Gene	Case subjects (*n* = 5538)		Control subjects (*n* = 5665)	
	*N* of variants	*N* of variant carriers	*N* of variants	*N* of variant carriers
*BRCA1*	33	44	0	0
*BRCA2*	53	65	1	1
*PALB2*	28	37	1	1
total	**114**	**142**	**2**	**2**

It has been well established that BCs from carriers of known pathogenic germline variants in *BRCA1*, *BRCA2* and *PALB2* almost universally show loss of the wildtype allele that is associated with characteristic genomic features such as numerous large scale copy number aberrations (measured by a HRD score) and specific somatic single base substitution (SBS) mutational signatures. It would be expected that genuine pathogenic non-coding variants would also be associated with these tumor genomic features, and this can be used to further prioritize the non-coding variants.

Formalin-fixed paraffin-embedded (FFPE) diagnostic blocks were obtained for 142 tumor samples carrying 114 prioritized variants. Of the four variants with SpliceAI scores ≥0.5, two were already included among these 114 prioritized variants, and FFPE blocks were additionally retrieved for the remaining two cases. Accordingly, all 116 variants (114 prioritized plus two additional SpliceAI-predicted variants) are included in Table S5.

Of the 116 high priority variants (144 cases, prioritized subset) with at least one tumor identified, 42 variants (tumors WGS available subset) could be assessed using 40 tumor samples from 40 patients, as some archival specimens had been discarded or had been exhausted in prior analyzes. Sufficient quantity and quality of tumor DNA was obtained from 40 carriers, enabling completion of WGS. This cohort included 14 *BRCA1* cases with 15 variants, 15 *BRCA2* cases with 16 variants, and 11 *PALB2* cases with 11 variants (Table 5, and Table S6). The variant allele fraction (VAF) was assessed for each location to determine if there was allele loss at that position described previously^14^.

**Table 5:** Summary of all sequenced *BRCA1*, *BRCA2* and *PALB2* non-coding region variants

Variants	Gene	cDNA change^a^	*N*^o^ Case	*N*^o^ Control	Sequenced tumor^b^	Specific variant allele status^c^	HRD Level^d^	Functional Impact^e^	Note
chr17: g.43049663 A > T	*BRCA1*	c.5333-469 T > A	2	0	1	HET	Low	U	
chr17: g.43058247 G > A	*BRCA1*	c.5194-1112 C > T	1	0	1	LOH (loss of WT)	Moderate	FI	
chr17: g.43061104 G > A	*BRCA1*	c.5193+2229 C > T	2	0	1	HET	High	U	
chr17: g.43061618 T > C	*BRCA1*	c.5193+1715 A > G	2	0	1	HET	Low	U	
chr17: g.43062058 T > C	*BRCA1*	c.5193+1275 A > G	1	0	1	LOH (loss of WT)	High	FI	
chr17: g.43065747 T > C	*BRCA1*	c.5075-1796 A > G	2	0	1	LOH (loss of variant)	Moderate	FN	
chr17: g.43075220 T > C	*BRCA1*	c.4485-699 A > G	2	0	1	LOH (loss of variant)	High	FN	
chr17: g.43077207 A > G	*BRCA1*	c.4358-593 T > C	2	0	1	Not determined	Not determined	NA	
chr17: g.43082398 A > G	*BRCA1*	c.4357+6 T > C	1	0	1	LOH (loss of WT)	High	FI	Has been reported as a pathogenic variant
chr17: g.43086393 C > T	*BRCA1*	c.4186-3818 G > A	2	0	1	LOH (loss of WT)	High	FI	
chr17: g.43105000 C > T	*BRCA1*	c.213-44 G > A	2	0	1	HET	Moderate	U	
chr17: g.43113758 T > C	*BRCA1*	c.134+1968A > G	3	0	1	LOH (loss of WT)	High	FI	
chr17: g.43119136 G > T	*BRCA1*	c.81-3357 C > A	2	0	1	LOH (loss of variant)	High	FN	
chr17: g.43119581 T > C	*BRCA1*	c.81-3802 A > G	1	0	1	LOH (loss of variant)	High	FN	
chr17: g.43125025 T > C	*BRCA1*	c.1-929 A > G	2	0	1	Not determined	Not determined	NA	
chr13: g.32321874 C > A	*BRCA2*	c.316+2549 C > A	3	0	1	LOH (loss of WT)	Moderate	FN	Carrier with a HET somatic *BRCA2* frameshift mutation
chr13: g.32328185 T > A	*BRCA2*	c.632-1258 T > A	1	0	1	HET	Low	U	
chr13: g.32345846 A > C	*BRCA2*	c.6938-981 A > C	1	0	1	LOH (loss of WT)	High	FI	
chr13: g.32347616 T > C	*BRCA2*	c.7007+720 T > C	1	0	1	HET	High	U	
chr13: g.32350779 T > C	*BRCA2*	c.7007+3883 T > C	3	0	1	HET	High	U	
chr13: g.32357056 A > T	*BRCA2*	c.7617+447 A > T	2	0	1	LOH (loss of variant)	Low	FN	
chr13: g.32357148 C > A	*BRCA2*	c.7617+539 C > A	3	0	1	HET	Moderate	U	Carrier with a HET somatic *BRCA2* frameshift mutation
chr13: g.32364585 A > G	*BRCA2*	c.8331+1052 A > G	4	0	2	LOH (loss of variant)	High	FN	
						LOH (loss of variant)	High		
chr13: g.32364658 A > G	*BRCA2*	c.8331+1125 A > G	1	0	1	LOH (loss of variant)	Moderate	FN	
chr13: g.32369221 T > C	*BRCA2*	c.8332-1181 T > C	1	0	1	LOH (loss of WT)	High	FI	
chr13: g.32377771 A > G	*BRCA2*	c.8754+980 A > G	2	0	1	HET	Low	U	
chr13: g.32383426 G > A	*BRCA2*	c.9256+3281 G > A	1	0	1	HET	High	U	
chr13: g.32386549 T > C	*BRCA2*	c.9256+6404 T > C	1	0	1	LOH (loss of WT)	High	FI	
chr13: g.32387654delGTCACACTCCTT	*BRCA2*	c.9257-7031_9257-7020del12	1	0	1	LOH (loss of WT)	High	FI	
chr13: g.32394936 A > T	*BRCA2*	c.9501+3 A > T	3	0	1	LOH (loss of WT)	Low	FN	
chr13: g.32396248 A > C	*BRCA2*	c.9502-650 A > C	5	0	1	HET	Moderate	U	Carrier with a HET somatic *BRCA2* frameshift mutation
chr16: g.23604049 C > T	*PALB2*	c.3351-380 G > A	3	0	1	LOH (loss of WT)	High	FI	
chr16: g.23605469 C > T	*PALB2*	c.3351-1800G > A	4	0	1	HET	High	U	
chr16: g.23608408 G > A	*PALB2*	c.3202-396 C > T	6	0	2	LOH (loss of WT)	Low	FN	
						LOH (loss of variant)	Moderate		
chr16: g.23614330 T > A	*PALB2*	c.3114-239 A > T	3	0	1	LOH (loss of WT)	High	FI	
chr16: g.23622220 G > A	*PALB2*	c.2997-742 C > T	3	0	2	LOH (loss of variant)	High	FN	
						HET	Moderate		
chr16: g.23623141 T > C	*PALB2*	c.2835-11 A > G	3	0	2	LOH (loss of variant)	Low	FN	
						Not determined	Not determined		
chr16: g.23633163 G > T	*PALB2*	c.1684+1699 C > A	5	0	1	HET	High	U	
chr16: g.23636918 C > T	*PALB2*	c.212-584 G > A	5	0	1	HET	High	U	
chr16: g.23637483 A > T	*PALB2*	c.211+367 T > A	3	0	1	HET	Low	U	
chr16: g.23638328 A > G	*PALB2*	c.49-199 T > C	5	0	1	HET	High	U	
chr16: g.23643092 G > A	*PALB2*	c.117+69 C > T	3	1	2	HET	High	U	
						HET	High		

^a^The cDNA changes were determined based on the MANE transcripts: *BRCA1* (NM_007294.4), *BRCA2* (NM_000059.4), and *PALB2* (NM_024675.4).

^b^Tumor counts do not reflect the number of unique carriers, as individual tumors may contain multiple variants.

^c^Detailed the status of each candidate variant in the tumor. “LOH” indicates loss of heterozygosity, specifying whether the wild type or mutant allele was lost; heterozygous (HET); or where tumor purity was insufficient for reliable assessment (Not determined).

^d^HRD level was classified as High, Moderate, or Low based on genomic profiling.

^e^Functional Assessment categories: “FI”, Potential Functional impact; “FN”, Likely Functional neutral; “U”, Uncertain; “NA”, not assessable because of poor sequencing quality/depth.

As summarized in Table 6, 12 of the 42 variants showed loss of the wild-type allele and were in tumors with moderate or high HRD scores with *BRCA1* showing the highest proportion of variants with these associations (5 variants out of 15 total, 33%). The two SpliceAI predicted variants (*BRCA1* c.5194-1112 C > T and *PALB2* c.3114-239 A > T) both showed loss of the wild type allele and were in tumors with moderate/high HRD scores. Of the 12 variants initially meeting the LOH and HRD criteria, one *BRCA2* variant (chr13:g.32321874 C > A, c.316+2549 G > T) from sample Case-695 was subsequently excluded. Although this variant showed loss of the wild-type allele and occurred in a tumor with a high HRD score, somatic analysis of the same sample identified a heterozygous frameshift *BRCA2* mutation (chr13:g.32363250delC; c.8048delC; p.A2683Efs*11) with a CADD score of ~25. Given the uncertainty as to whether the observed HRD was attributable to the non-coding variant or the somatic mutation, this case was removed from the final count.

**Table 6:** Summary of non-coding variant allele status and tumor HRD score

Gene	*N*^o^ variants assessed	Variant allele status			Moderate/high HRD score + wild-type allele loss^a^
		Heterozygous	Variant loss	Wild-type loss	
*BRCA1*	15	4	4	5	5 (33%)
*BRCA2*	16	7	3	6	5 (32%)
*PALB2*	11	6	3	2	2 (15%)

^a^One BRCA2 variant was excluded due to the presence of a BRCA2 somatic frameshift mutation.

Where the tumor remained heterozygous for non-coding variants, and no somatic genetic variants was identified in the respective gene, methylation sequencing was performed; no evidence of promoter hypermethylation was detected in any tumor. Methylation analysis was also conducted on cases with moderate to high HRD scores and loss of the wild-type allele to eliminate the possibility that loss of HR function was not due to promoter hypermethylation (rather than the observed non-coding variant); again, no promoter hypermethylation was detected.

Overall, across the 42 variants assessed, 11 variants were considered to have features consistent with functional impact and potentially pathogenicity.

### Blood RNA sequencing of carriers of potentially pathogenic non-coding variants

For these 11 potentially pathogenic variants, fresh blood samples were sought from all available carriers for transcript analysis. Ultimately, blood samples were obtained from four carriers representing four variants: *BRCA1* c.134+1968A > G, *BRCA2* c.8332-1181 T > C, *BRCA2* c.9256+6404 T > C, and *PALB2* c.3351-380 G > A. RNA sequencing was performed on peripheral blood lymphocytes. However, expression levels of the corresponding genes in blood were low, precluding meaningful assessment of aberrant splicing or other transcript alterations associated with these variants.

### Functional assessment of candidate pathogenic non-coding variants

Functional assessment of pathogenicity was performed for the two variants (chr17:g.43058247 G > A and chr16:g.23614330 T > A) predicted to create deep intronic splicing sites, using CRISPR/Cas9 genome editing of the normal breast epithelial cell line MCF10A. The four carriers of these two variants were unrelated and ascertained independently. The SpliceAI-10k calculator^15^ predicted both variants to result in pseudoexon activation, with chr17:g.43058247 G > A generating a 118 bp pseudoexon and chr16:g.23614330 T > A generating a 161 bp pseudoexon, each introducing a premature stop codon into the predicted aberrant transcripts. A homozygous knock-in clone was established for the *BRCA1* variant chr17:43058247 G > A (c.5194-1112 C > T), and a heterozygous knock-in clone was established for *PALB2* variant chr16:23614330 T > A (c.3114-239 A > T). In addition, extra *BRCA1* knock-in clones were developed targeting the region surrounding the chr17:43058247 variant. This clone had a homozygous deletion (chr17:43058244_43058261del; c.5194-1126_5194-1109del18), which removed the predicted splice site created by the variant. All edited clones were screened to confirm the absence of additional homozygous or heterozygous mutations within *BRCA1* and/or *PALB2*.

**Fig. 3:**
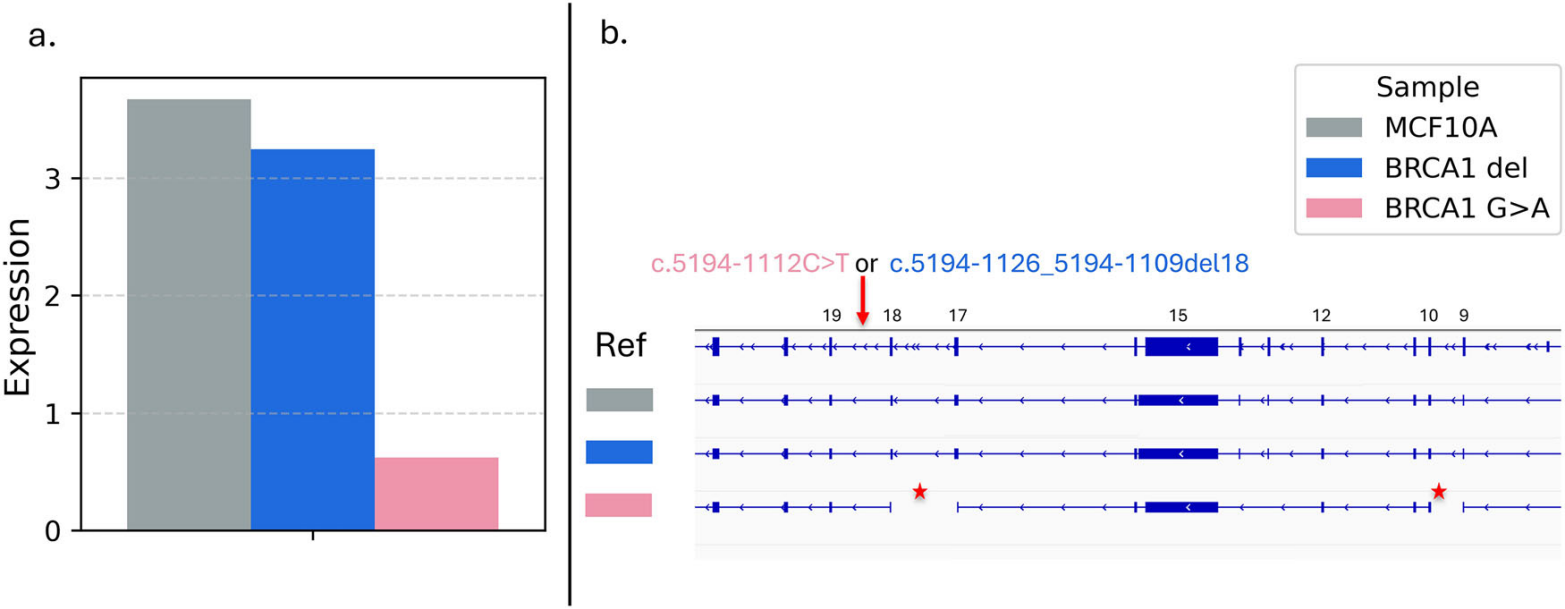
*BRCA1* expression in *BRCA1* knock-in clones assessed by RNA sequencing. **a** Relative *BRCA1* expression levels in the knock-in clone carrying the intended non-coding variant (c.5194-1112 C > T, pink), the clone with a deletion spanning the knock-in site (c.5194-1126_5194-1109del18, blue), and control MCF10A cells (grey). The Y-axis represents *BRCA1* transcript expression levels quantified as transcripts per million (TPM), calculated from FeatureCounts-derived read counts after normalization for transcript length and sequencing depth. **b** RNA-seq visualization of *BRCA1* transcript structures. The reference transcript is shown at the top, followed by MCF10A, the c.5194-1126_5194-1109del18 clone (BRCA1 del), and the knock-in (c.5194-1112 C > T) clone (BRCA1 G > A). Red arrows indicate the location of the knock-in site, and red asterisks denote regions of transcript disruption.

**Fig. 4:**
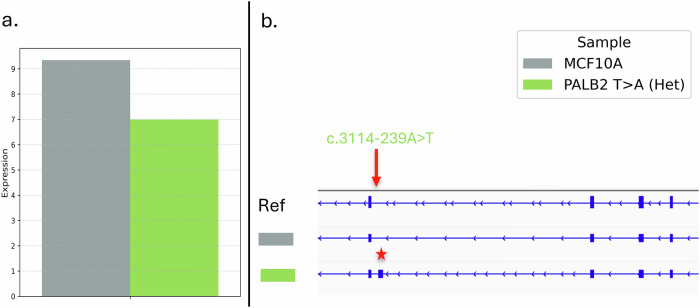
*PALB2* expression in *PALB2* knock-in clones assessed by RNA sequencing. **a** Relative *PALB2* expression levels in the knock-in clone carrying the intended non-coding variant (c.3114-239 A > T, green), compared to control MCF10A cells (grey). The Y-axis represents *PALB2* transcript expression levels quantified as transcripts per million (TPM), calculated from feature counts-derived read counts after normalization for transcript length and sequencing depth. **b** RNA-seq visualization of *PALB2* transcript structures. The reference transcript is shown at the top, followed by MCF10A as control and the knock-in (c.3114-239 A > T) clone. The red arrow indicates the location of the knock-in site, and the red asterisk marks a region of transcript disruption.

RNA sequencing of the *BRCA1* clones revealed markedly reduced *BRCA1* transcript levels in the non-coding variant knock-in clone (chr17:43058247 G > A; c.5194-1112 C > T), relative to the parental MCF10A cells (Fig. 3). In contrast, the *BRCA1* knock-in clone that removed the non-coding variant location showed similar *BRCA1* expression levels to the wild-type MCF10A cells. For the *PALB2* heterozygous knock-in clone, no substantial reduction in *PALB2* transcript abundance was observed. However, transcriptome analysis indicated the presence of an aberrant transcript incorporating a short-inserted sequence (161 bp) located three base pairs downstream of the knock-in site (Fig. 4).

## Discussion

Over the past three decades, the search for HBC genes has yielded significant breakthroughs, most notably the identification of *BRCA1* and *BRCA2* in the 1990s and, subsequently, *PALB2*, which together account for a substantial proportion of HBC. However, progress in identifying additional major hereditary BC susceptibility genes has been limited, with only a few moderate-risk genes, principally *CHEK2* and *ATM*, being established since then. Very high genetic heterogeneity of HBC is likely to partly explain the slow advance but data from the BEACCON study suggests that individual novel genes are unlikely to contribute a large proportion of remaining HBC cases, with similar findings from other international studies^16,17^. Consequently, previous work from our group shifted towards exploring alternative mechanisms of inactivation of known HBC genes^7^, notably estimating the proportion of pathogenic variants that may lie within the non-coding regulatory regions of *BRCA1*, *BRCA2* and *PALB2*.

In this study, we conducted a systematic case-control exploration of the non-coding regions in *BRCA1*, *BRCA2* and *PALB2* involving women with a diagnosis of HBC but no pathogenic variant identified in a known HBC gene by standard clinical genetic testing.

We have shown that for all three genes, there was a significant excess of rare-non-coding variants among the 5770 HBC cases and 5741 controls. As expected for these genes which are all associated with TNBC (to varying degrees), the excess of rare non-coding variants was greater among ER negative or TNBC cancer cases, particularly *BRCA1* (*p* = 0.0001). These data support the presence of pathogenic non-coding variants within the group of cases carrying rare variants; additionaly the modest effect size suggests that most rare non-coding variants are likely neutral, and pathogenicity must be assessed at the level of individual variants using additional lines of evidence. Exploiting what is known about the genomic consequences of known germline pathogenic variants in *BRCA1*, *BRCA2* and *PALB2* coupled with access to BC biospecimens from participants of the BEACCON study, we demonstrated that approximately 26.2% of sequenced cases had features consistent with being pathogenic based on loss of the wild-type allele and the tumor showing moderate or high HRD scores.

Among the 11 variants categorized as potentially pathogenic, one *BRCA1* variant (chr17:g.43082398 A > G) was not initially captured by our prioritization process. This variant was listed in ClinVar with conflicting interpretations (Pathogenic/Uncertain), and its SpliceAI score was low (0.08), providing little predictive support for a splicing effect. Nevertheless, the carrier presented with TNBC and a strong family history of related cancers. More recently, a study from Baralle lab employing RNA sequencing demonstrated that this variant causes a splicing aberration resulting in r.4185_4356del, p.(Arg1397TyrfsTer2)^18^, thereby confirming its pathogenicity. This result is concordant with our tumor sequencing findings and reinforces the validity of our prioritization framework for identifying potentially pathogenic non-coding variants.

Two potentially pathogenic variants, *BRCA1* c.5194-1112 C > T and *PALB2* c.3114-239 A > T, were functionally assessed using CRISPR knock-in in the MCF10A cell line. These two variants were identified in a total of four carriers; however, blood samples were not available from any of these individuals, and RNA sequencing of patient-derived lymphocytes could therefore not be performed. Nevertheless, the engineered mutant cell lines demonstrated a marked reduction in transcript expression and clear disruption of normal transcript processing, consistent with SpliceAI predictions, providing strong functional evidence of pathogenicity. The fragmented abnormal *BRCA1* transcripts observed in RNA sequencing may reflect instability of aberrantly spliced transcripts and their partial degradation, potentially consistent with nonsense-mediated decay or related RNA surveillance mechanisms, although the precise underlying mechanism remains to be clarified. Interestingly, an additional *BRCA1* knock-in clone that carried an 18 bp deletion that removed the non-coding variant exhibited normal transcript levels reinforcing the conclusion that the splicing defect is specific to the chr17:g.43058247 G > A variant and not attributable to disruption of a broader regulatory element.

Identification of the *PALB2* non-coding variant (chr16:g.23614330 T > A; c.3114-239 A > T) as potentially pathogenic is consistent with a recent study using long read sequencing that shows this variant creates a pseudoexon containing a premature stop codon that is detected in peripheral blood monocyte RNA from a woman with ER + BC diagnosed at age 22^9^. The genomic coordinates of this previously detected pseudoexon were concordant with the SpliceAI-10k calculator prediction for *PALB2* c.3114-239 A > T, which also aligned with the results of our sequencing analysis of the *PALB2* knock-in clone. Collectively, these findings support the accuracy and practical value of machine-learning–based splicing prediction tools, such as SpliceAI and the SpliceAI-10k calculator, in identifying non-coding variants with potential functional consequences. At the same time, the RNA sequencing evidence from the Baralle group illustrates that in silico predictions are not infallible. This highlights the need to integrate multiple lines of evidence, including clinical features, and personal and family cancer histories, when assessing the pathogenicity of non-coding variants.

To evaluate the maximum potential contribution on splice-altering non-coding variants to HBC, we compared the numbers of predicted splice-altering variants using a lower SpliceAI cutoff. Even when applying the more permissive threshold of 0.2, only 15 unique variants (among 19 cases) with potential splice-altering effects were identified suggesting the proportion of familial BC cases that can be recognized by current tools as splice-altering non-coding variants is small and not exceeding about 0.3% of the full case cohort.

This study provides evidence for the existence of additional penetrant pathogenic non-coding variants in *BRCA1*, *BRCA2*, and *PALB2* but estimating the overall contribution to HBC is difficult to ascertain. We have shown that ~50% of HBC families in the BEACCON study harbour at least one rare non-coding variant. Among the subset of families for which tumor WGS data were available, 26.2% (11/42) of whole genome sequenced variants exhibited genomic features consistent with pathogenicity. Taken together, these findings suggest that rare non-coding variants may account for up to approximately 10% of currently unexplained HBC cases in the full case cohort. This estimate aligns closely with previous analyzes that used a maximum likelihood approach on the BEACCON case–control data that assumed benign variants occur equally in cases and controls and that pathogenic variants confer risk consistent with established odds ratios^7^. While the case–control design provides strong evidence for the existence of a substantial number of pathogenic non-coding variants, this study also highlights the inherent difficulty of confirming pathogenicity for any single variant, even when supported by tumor sequencing data and functional modelling.

Despite the insights into the contribution of non-coding variants to HBC this study has certain limitations. Our highly selective filtering to identify candidate pathogenic non-coding variants may have overestimated the contribution of non-coding variants towards pathogenicity for unexplained HBC. Conversely many potential variants were excluded based on their detection in cases with ER + BC which is a strong but not exclusive predictor of pathogenicity, particularly for *BRCA2* and *PALB2*.

Additionally, HRD classification in this study was informed primarily by CNVkit-derived copy number profiles, with HRD scores used as supportive rather than definitive evidence. We observed variability in HRD score outputs when different computational tools were applied to the same FFPE sequencing data, sometimes yielding uniformly high scores inconsistent with CNV patterns. This combined approach may introduce some false-positive HRD classifications; however, applying the conventional HRD threshold of ≥42 would have excluded the two experimentally validated knock-in variants, both of which had HRD scores below this cutoff despite independent evidence supporting likely pathogenicity. Further work is needed to optimize HRD scoring approaches for FFPE samples.

In conclusion, full-gene, large-scale sequencing in *BRCA1*, *BRCA2* and *PALB2* among HBC cases without identifiable pathogenic variants in known HBOC genes supports the presence of pathogenic non-coding variants within intronic and promoter regions as one possible explanation for disease presentation in a subset of cases. Given the rarity of individual non-coding variants, validating their pathogenicity through clinical approaches such as segregation analysis is challenging. Robust evaluation will require in vitro functional studies, together with assessment of gene expression and transcript integrity in primary biospecimens from variant carriers.

## Methods

### Case cohort and controls

The cases and controls were drawn from the BEACCON study and have been described in detail elsewhere^6^. Briefly, the 5,770 cases comprised index patients diagnosed with female breast and/or ovarian cancer drawn from the Variants in Practice Study (*n* = 3065) and the Hunter Area Pathology Service, Newcastle, Australia (*n* = 2705). All cases underwent assessment at specialist Familial Cancer Clinics and met the eligibility criteria for clinical genetic testing of hereditary breast and ovarian cancer (HBOC) genes (≥10% probability of a pathogenic variant) but tested negative for *BRCA1*, *BRCA2* and *PALB2* pathogenic variants^6^.

The control group consisted of 5,741 cancer-free female subjects aged over 40 years, recruited from the Lifepool Study^19^. The mean age at diagnosis for cases was 49.7 years (range: 19.0–94.8), and 65.6 years (range: 40.0–97.5)^6^ for controls, the older unaffected control group was selected to minimize the likelihood of future cancer diagnoses, ensuring the highest chance that they represented the ture cancer-free individuals. Cases and controls were predominantly of European ancestry (>90%), with comparable ancestry composition between cohorts as detailed in the previously reported data from the BEACCON study^6^. This study received approval from The Human Research Ethics Committee at The Peter MacCallum Cancer Centre (Approval # 09/29) and all participating centers, with continued matching to cancer registries to exclude incident cancer diagnoses. All participants provided informed consent for genetic analysis of their germline DNA.

### Germline sequencing of non-coding regions

Sequencing data from a total of 11,511 individuals (5770 breast cases and 5741 controls) was analyzed to identify rare variants in the non-coding regions and 5’ upstream promoter regions of *BRCA1, BRCA2* and *PALB2*. The full locus and 5’ upstream region of *BRCA1* (chr17: 43044294 - 43145483, hg38; including 20 kb upstream), *BRCA2* (chr13: 32295479 - 32399672, hg38; including 20 kb upstream) and *PALB2* (chr16: 23603160 - 23646357, hg38; including 5 kb upstream) were sequenced as reported in the BEACCON study^6^. All sequencing was performed by the Australian Genome Research Facility (AGRF), with case and control samples processed concurrently and distributed across sequencing batches to minimize potential batch effects.

### Variant filtering

A series of filtering steps were applied to identify rare non-coding variants in *BRCA1*, *BRCA2*, and *PALB2* that were more likely to be pathogenic. Variants were first screened to remove potential sequencing artefacts. For recurrent variants observed in multiple samples, inclusion was determined based on sequencing quality metrics; variants were retained if the majority of occurrences passed the quality filters.

Quality control thresholds were defined based on the distribution of variant allele fraction (VAF). Examination of the VAF distribution showed a negatively skewed bimodal pattern, with genuine heterozygous germline variants clustering around ~0.5 and artefacts near 0. To determine an appropriate lower threshold, the distribution of VAF values between 0.1 and 1 was analyzed (Fig. S1), and the rate of change in the distribution was evaluated using first- and second-derivative analyzes to identify an inflection point (Fig. S1). This analysis indicated an inflection point at approximately 0.26, which was therefore used as the lower VAF threshold. An upper threshold of 0.75 was applied to exclude variants inconsistent with the expected heterozygous state of germline pathogenic variants in these genes under the two-hit model. Variants were therefore retained if 0.26 ≤ VAF ≤ 0.75.

Sequencing depth filtering was also applied, with a minimum sequencing depth threshold selected to retain approximately 90% of sequencing observations (Fig. S2). Individual variants were removed if their maximum observed allele frequency across all populations in 1000 Genomes, ESP, and gnomAD (v4.1.0, https://gnomad.broadinstitute.org/) population control databases exceeded 0.0005. In addition, using the genetic ancestry group frequency data in gnomAD (v4.1.0), variants were excluded if the highest sub-population allele frequency exceeded 0.01.

### Robustness analysis of variant filtering

To assess the robustness of the association between rare non-coding variants and case–control status, repeated internal cross-validation analyzes were performed. Across 10 independent iterations, the combined case–control dataset was randomly partitioned into two approximately equal subsets while preserving the overall case–control proportions.

For each subset, the full variant filtering and analytical pipeline was reapplied independently, including exclusion of individuals harbouring pathogenic or likely pathogenic exonic variants in known HBOC genes, application of sequencing quality filters (variant allele fraction 0.26–0.75 and sequencing depth ≥26), and population allele frequency filtering using the 1000 Genomes, ESP, and gnomAD (v4.1.0) databases.

Odds ratios (ORs) and 95% confidence intervals were calculated for each subset using the same statistical framework as in the primary analysis. Consistency of effect estimates across cross-validation subsets was assessed by examining: direction consistency (proportion of subsets with OR > 1); statistical significance consistency (proportion with *P* < 0.05); distribution of OR estimates across subsets (median, interquartile range and range), and agreement between paired halves within each iteration based on differences in log-transformed ORs. Summary statistics from these analyzes are provided in Table S7.

### Analysis of potential splicing impact

All variants passing both sequencing quality and rarity filters were evaluated for their potential impact on RNA splicing using SpliceAI (v1.3.1)^20^.

For all analyzes, SpliceAI^20^ was run on GRCh38 with the default model using a GTF restricted to the MANE transcripts for *BRCA1* (NM_007294.4), *BRCA2* (NM_000059.4) and *PALB2* (NM_024675.4) downloaded from UCSC; to maximize capture of potential splicing impact, the maximum distance between a variant and a gained/lost splice site was 4999 bp. Variant prioritization was performed using both the SpliceAI-recommended high-impact threshold of 0.5, and a more permissive threshold of 0.2^20^, the latter of which is recommended by the ClinGen Sequence Variant Interpretation Splicing Subgroup^13^. Additionally, variants classified as Benign in ClinVar (https://www.ncbi.nlm.nih.gov/clinvar/) were excluded irrespective of SpliceAI score. Variants with SpliceAI scores < 0.2 in gnomAD (v4.1.0; using SpliceAI annotations provided within the database, which are based on slightly different parameters from those used in our primary SpliceAI analysis) were also excluded. Final statistics and downstream prioritization were based on these stratified SpliceAI predictions.

In addition to using SpliceAI to assess potential splicing impact, SpliceAI-10k calculator was used to predict variant-induced splicing alterations^15^. All analyzes were performed with default parameters.

### Prioritization of case carriers for tumor sequencing

To prioritize carriers of non-coding variants for tumor sequencing, we applied a set of criteria incorporating case–control enrichment, tumor phenotype, and family cancer history. Because family history data were only available for carriers from the Variants in Practice (ViP) Study, family-history–based filtering was restricted to these samples. For variants observed in multiple carriers, retention required that the majority of carriers exhibited a relevant family history of cancer.

Variant carriers were prioritized based on three principal features: (i) elevated case–control enrichment, defined by gene-specific odds ratio thresholds; (ii) tumor phenotype, particularly TNBC in the proband; and (iii) family cancer history, quantified using predefined family history scores reflecting multi-generational occurrences of relevant cancers.

For *BRCA1* carriers, prioritization considered variants with OR ≥ 4, TNBC status, and a family history score ≥4 reflecting multi-generational breast and/or ovarian cancer (Fig. S3). For *BRCA2* carriers, prioritization included variants with OR ≥ 6, TNBC status, and family history features defined by the presence of male breast or prostate cancer among relatives (Fig. S4). For *PALB2* carriers, prioritization considered OR ≥ 4, TNBC status, and family history of breast or pancreatic cancer in close relatives (Fig. S5).

The overlap and distribution of these prioritization criteria were visualized using UpSet analysis, enabling identification of carriers fulfilling single or multiple risk categories. Samples meeting any of these criteria were retained for downstream analyzes, with highest priority assigned to those fulfilling multiple conditions.

### Tumor whole genome sequencing and variant calling

Breast tumors and matched blood-derived germline DNA from patients carrying potential pathogenic non-coding variants underwent whole-genome sequencing (WGS). The matched germline samples were used as references for somatic variant calling. Downstream analyzes used two complementary data layers. The zygosity of each candidate variant (heterozygous, loss of the wild-type allele or loss of the variant allele) was inferred from the tumor WGS data based on variant allele fraction and local copy-number context, whereas homologous recombination deficiency (HRD) and mutational signatures were assessed from the catalogue of somatic variants obtained from paired tumor–normal calling. Tumor DNA was extracted from cancer cells in formalin-fixed, paraffin-embedded (FFPE) slides by needle micro-dissection using the QIAamp DNA FFPE Tissue Kit (Qiagen, CA, USA) as described previously^21^. WGS was conducted by the Australian Genome Research Facility (AGRF, https://www.agrf.org.au/). DNA libraries were prepared using the xGen™ cfDNA & FFPE DNA Library Preparation Kit. Sequencing was performed to achieve 30X coverage (approximately 100Gbp of raw data) on a NovaSeq 6000 platform (v1.5 chemistry). Sequence alignment was carried out using BWA (v0.7.17) algorithm, and variant calling was performed with GATK (v4.0.4.0).

### Determination of variant allelic status

Biallelic inactivation of a germline heterozygous variant was determined from the tumor VAFs, adjusted according to tumor purity (TP) estimates confirmed by manual inspection and CNVkit analysis as we described previously^22^. In addition, the sequencing data were assessed for any somatic potential loss of function variants in *BRCA1*, *BRCA2* or *PALB2*.

### CNV analysis, HRD score calculation

Copy number plots were generated for each tumor using CNVkit (v0.9.9)^23^, with visualization accomplished through custom R (v4.0.2) scripts. HRD scores were calculated for each tumor sample based on the CNVkit copy number results, and an HRD score computed by using scarHRD^24^ as the sum of the occurrence of telomeric allelic imbalances^25^, large-scale state transitions^26^, and HR deficiency-loss of heterozygosity^27^. Although a threshold of 42 is commonly employed to identify high HRD, this measure may be less reliable in FFPE samples. Consequently, a threshold HRD scores of >28 was used.

### Somatic variants, mutational signature analysis and HRDetect score calculation

Somatic variants were identified using GATK Mutect2 (v4.1.4.1) through joint calling of tumor and matched germline WGS data. Somatic variants were then filtered according to the following criteria: sequencing depth of tumor variants (TUMOR: DP ≥ 15), allele frequency of tumor variants (TUMOR:AF ≥ 0.25), and population allele frequency (gnomAD_v4_MAX_AF < 0.01). To reduce the number of formalin fixation induced base changes in FFPE samples, FFPEsig (v3.0)^28^, a computational algorithm designed to identify and remove formalin-induced artefacts was applied to the somatic data. This correction step was applied to the FFPE WGS data prior to both mutational signature analysis and HRDetect score calculation.

Mutational signature analysis was conducted using signature.tools.lib (v2.4.5)^29^. To reduce data overfitting, only breast organ signatures were used. The visualization of the results was accomplished using a custom Python (v3.8.13) script.

### Promoter methylation sequencing and data analysis

Promoter hypermethylation was assessed for *BRCA1, BRCA2* and *PALB2* in tumor DNA from cases carrying a potential pathogenic non-coding variant using the Twist NGS Methylation Sequencing system targeting the full promoter region of each gene that included the regulatory CpG island. Tumor DNA was enzymatically deaminated using the NEBNext Enzymatic Methyl-seq Methylation Library Preparation Kit. Sequencing was performed to achieve 200X coverage (approximately 200Gbp of raw data) by NovaSeq 2×100 bp experiment. Raw sequencing data were processed using a standardized pipeline from Twist Bioscience (https://nf-co.re/methylseq/1.6.1). The reference genome was prepared by indexing with BWA (v0.2.2) and creating a dictionary file using Picard (v2.22.8). Initial quality control of FASTQ files was performed with FastQC (v0.11.9), followed by adapter and low-quality base trimming using Trim Galore (v0.4.4). Trimmed reads were aligned to the reference genome using BWA-Meth (v0.2.2), with alignment files processed and sorted using samtools (v1.9) and bismark (v0.22.3). Duplicate reads were marked, and quality metrics were collected with GATK (v4.1.8.1). Methylation calling was performed with MethylDackel (v0.5.3). The visualization of the results was accomplished using a custom R (v4.0.2) script.

### Blood RNA sequencing and data analysis

RNA sequencing was performed on blood lymphocytes for cases carrying potentially pathogenic non-coding variants. RNA was extracted from a dedicated blood draw. RNA libraries were prepared using the NEBNext rRNA Depletion Kit, and sequencing was conducted on the NextSeq 2000 Sequencing System.

The raw sequencing data were first quality-checked using FastQC (v0.11.6) to assess read quality metrics. Adapter trimming and filtering of low-quality bases were performed using Cutadapt (v2.1) with a quality threshold of 15 and a minimum read length of 50 base pairs. The cleaned paired-end reads were aligned to the human reference genome (GRCh37) using HISAT2 (v2.0.4) with SNP and transcript annotations. The resulting alignments were converted to BAM format using Samtools (v1.8) and sorted by coordinate order with Picard (v2.17.3). BAM files were indexed to facilitate downstream analyzes.

RNA sequencing generated a mean of 101.2 million purity-filtered reads per sample (range: 86.5–136.3 million). On average, over 21,000 genes were detected per sample, confirming broad transcriptomic coverage. Read alignment rates were consistently high across samples (mean 92.7%) with a low base mismatch rate (~0.2%). Estimated library sizes ranged from 54.5 to 67.3 million unique fragments, with duplication rates between 28.9% and 34.9%. All samples passed quality control checks. These metrics indicate high-quality RNA sequencing data suitable for downstream analyzes. Finally, FeatureCounts^30^ and StringTie^31^ were used to perform transcript reconstruction and statistics on the basis of the reads-level results.

### In vitro CRISPR/Cas9 variants knock-in cells construction and RNA extraction

CRISPR/Cas9 genome editing was utilized to introduce two potentially pathogenic non-coding variants via nucleofection into the MCF10A cell line derived from normal breast epithelial cells. These variants are located in the non-coding regions of the *BRCA1* and *PALB2* genes, respectively. MCF10A cells were cultured and passaged in accordance with the Brugge laboratory protocol (https://brugge.hms.harvard.edu/system/files/protocols/MediaRecipesForMCF10ACells.pdf), with specific conditions detailed in Table S1. Given the increased fragility of MCF10A cells post-nucleofection, the serum concentration in the growth medium was elevated from 5% to 10% to enhance cellular recovery and viability. All other components of the growth medium remained consistent with the standard formulation.

Bi-allelic knock-in constructs were generated by integrating CRISPR/Cas9 with electroporation-based Nucleofector technology (Lonza, Switzerland), conducted at the Victorian Centre for Functional Genomics (VCFG), Peter MacCallum Cancer Centre, Victoria, Australia. Single-guide RNAs (sgRNAs) were designed utilizing the CRISPOR online tool (https://crispor.gi.ucsc.edu/), with specific sequences listed in the Table S2. The single-stranded DNA (ssDNA) donor templates used for knock-in were designed using the donor design tool available from Horizon Discovery (Cambridge, UK); precise sequences are provided in the Table S2.

MCF10A cells were cultured at 37 °C with 5% CO₂ in DMEM/F12 medium supplemented with 5% horse serum (Gibco, Catalog number: 16050122), 20 ng/mL Human Epidermal Growth Factor (EGF) (Sigma-Aldrich, Catalog number: SRP3027), 0.5 mg/mL hydrocortisone (Sigma-Aldrich, Catalog number: H-0888), 100 ng/mL cholera toxin (Sigma-Aldrich, Catalog number: C-8052), and 10 µg/mL insulin (PMCC Pharmacy). To facilitate recovery following nucleofection, the serum concentration was increased from 5% to 10%, while all other media components remained unchanged.

Following nucleofection, cells were cultured for approximately one week until ~70% confluence. A fraction of the cells was used for Sanger sequencing to assess CRISPR editing efficiency, while the remaining cells were expanded. Single-cell clones were subsequently isolated by FACS sorting into 96-well plates, expanded, and screened by Sanger sequencing to identify clones carrying the desired variants.

Validated clones were used to generate baseline genomic DNA samples (T0). Cells were then maintained under standard culture conditions for 60 days, after which a second round of single-cell sorting was performed to generate subclones (T60). DNA extracted from T60 subclones, together with the corresponding T0 samples, was subjected to whole-genome sequencing to identify somatic mutations acquired during the culture period.

Genomic DNA was extracted from bulk cell populations comprising between one and five million cells using the QIAGEN DNeasy Blood and Tissue Kit (Qiagen, CA, USA) following the manufacturer’s recommended procedures. DNA concentrations were subsequently measured using the Qubit fluorometric quantitation assay (Invitrogen, MA, USA).

RNA intended for functional analyzes was isolated using the QIAGEN RNeasy Kit (Qiagen, CA, USA), adhering to the manufacturer’s guidelines. RNA concentrations were accurately quantified with the Qubit fluorometric quantitation assay (Invitrogen, MA, USA).

### RNA sequencing and data analysis for variant knock-in cells

RNA sequencing and subsequent data analysis for the variant knock-in cell lines were conducted using the same methodology as the RNA sequencing of blood samples. Sequencing yielded an average of 142.7 million purity-filtered reads per sample, with a range from 125.7 to 165.7 million. Mapping rates were uniformly high across all samples, with a mean value of 95.3%, and a low base mismatch rate of approximately 0.4%. The estimated library sizes varied from 45.0 to 53.9 million unique fragments. Duplication rates ranged between 45.2% and 50.4%. On average, approximately 20,400 genes were detected per sample, indicating broad and robust transcriptomic coverage. All samples met the quality control criteria.

## Supplementary information

Supplementary_figures

supplementary_tables_6

## Data Availability

The sequencing data generated in this study are not publicly available due to ethical and privacy restrictions but are available from the corresponding author upon reasonable request and with appropriate ethical approval.

## Code availability

Python script used for data analysis available at https://github.com/Qihong0925/rare-non-coding-variants-in-BRCA1-BRCA2-and-PALB2-.git. The Seqliner code is available separately from the R script at http://bioinformatics.petermac.org/seqliner/. All other publicly available code used during whole genome sequence data processing and variant calling are available via the links mentioned within the methods.
