# Supplementary material for: Investigating the contribution of rare non-coding variants in BRCA1, BRCA2 and PALB2 to hereditary breast cancer
Source: NPJ Breast Cancer. 2026 Apr 4;12:73. doi: 10.1038/s41523-026-00942-z (PMC13187058; doi:10.1038/s41523-026-00942-z)
Supplement: Supplementary file 1 — Supplementary_figures [file 41523_2026_942_MOESM1_ESM.pdf]

Supplementary Figures

a.

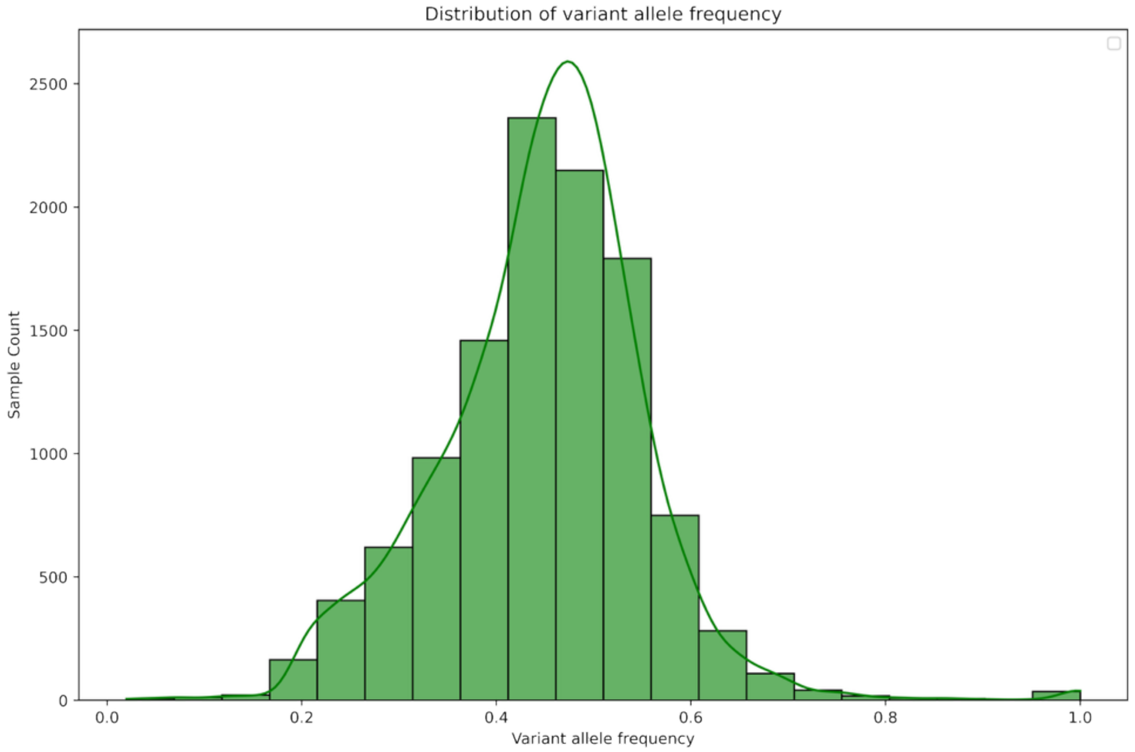

b.

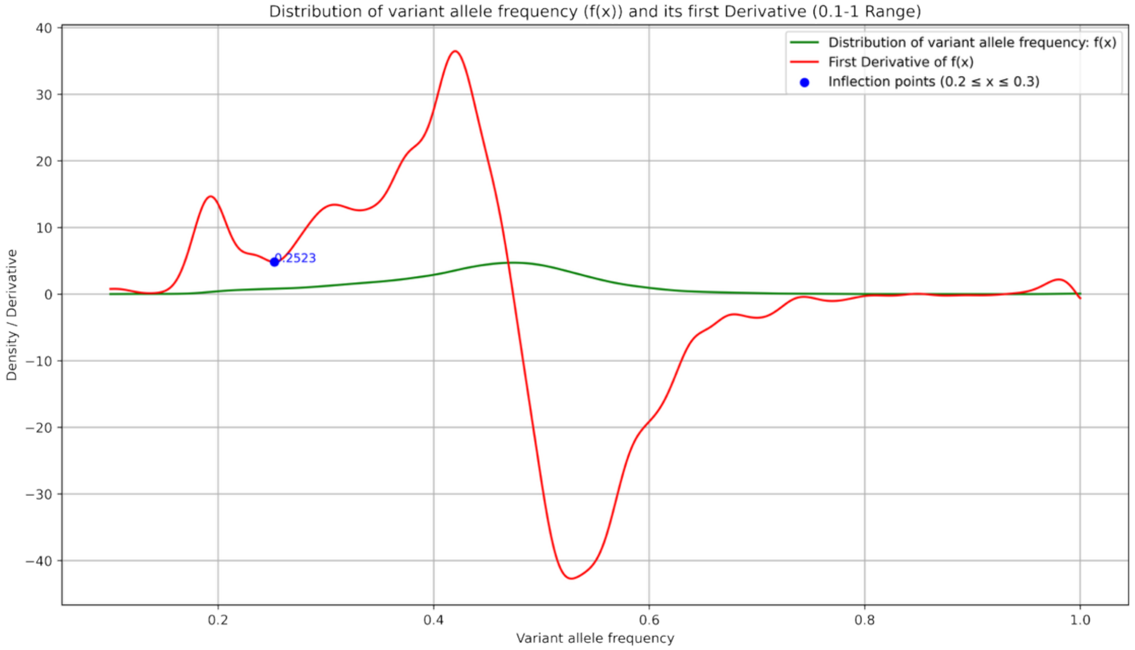

c.

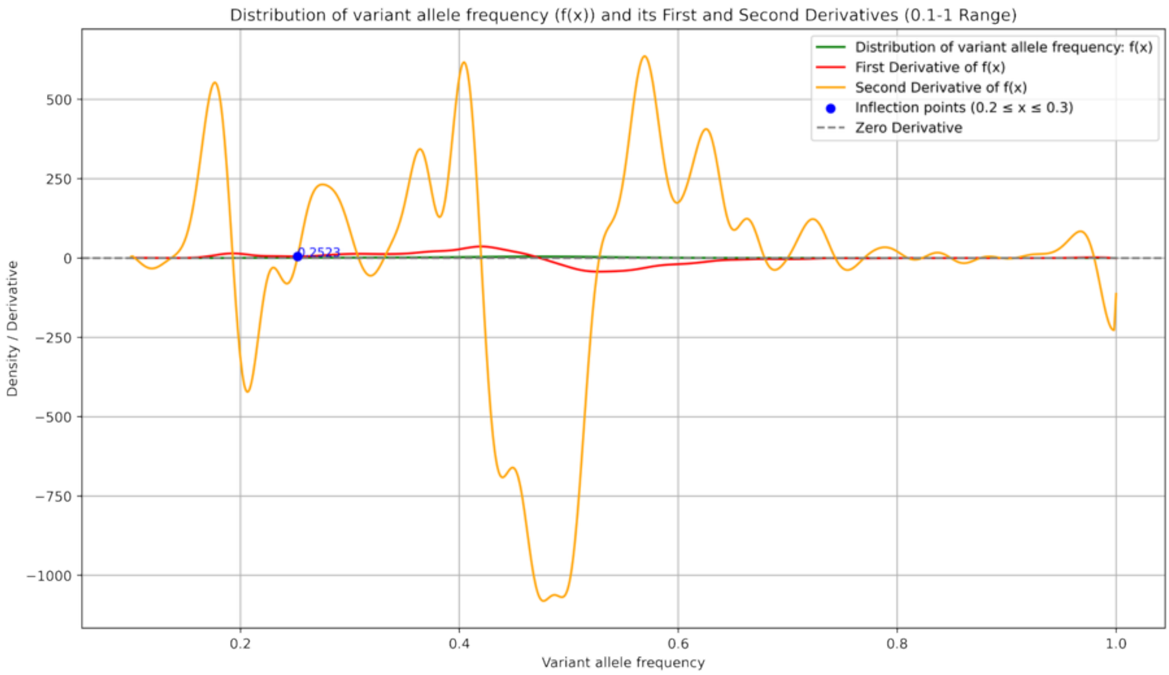

d.

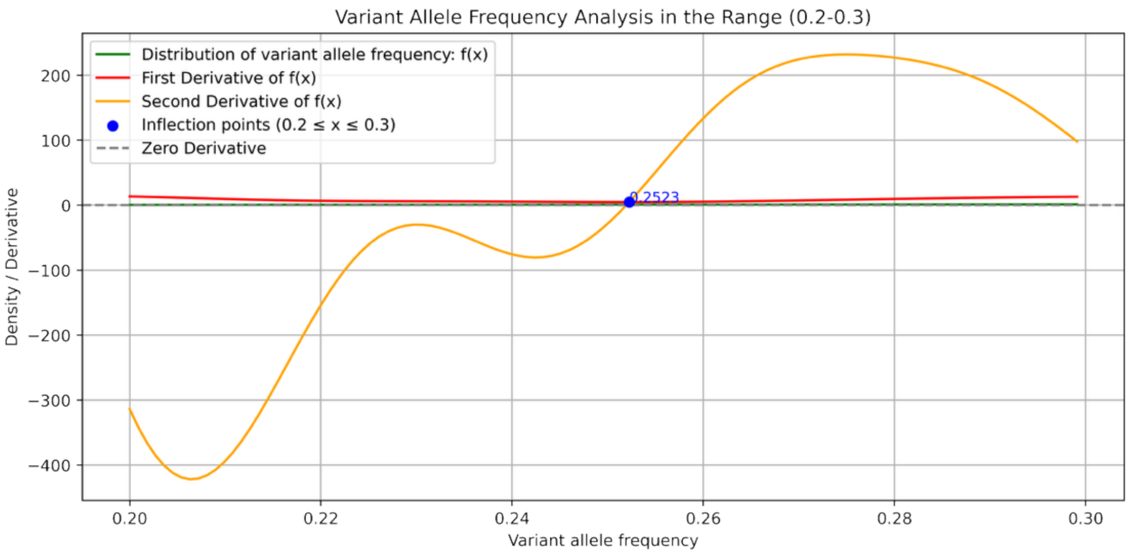

Figure S1. AF Distribution and Derivative Analysis for Non-coding BRCA1/2 and PALB2 Variants

(a) Histogram showing the distribution of variant allele frequencies (AF) for all identified non-coding *BRCA1*, *BRCA2*, and *PALB2* variants.

(b) Kernel density estimate of AF distribution (green line), with the first derivative (red line) plotted to visualize the rate of change in AF frequency across the cohort. The first derivative highlights local peaks and valleys, corresponding to changes in the density of observed AF values.

(c) The second derivative of the AF distribution, plotted alongside the first derivative and the original density (legend as indicated), enables identification of inflection points in the distribution.

(d) Zoomed-in view of the second derivative within the AF range of 0.2–0.3, highlighting inflection points (blue dot), which mark changes in the concavity of the distribution and may indicate biologically meaningful AF thresholds.

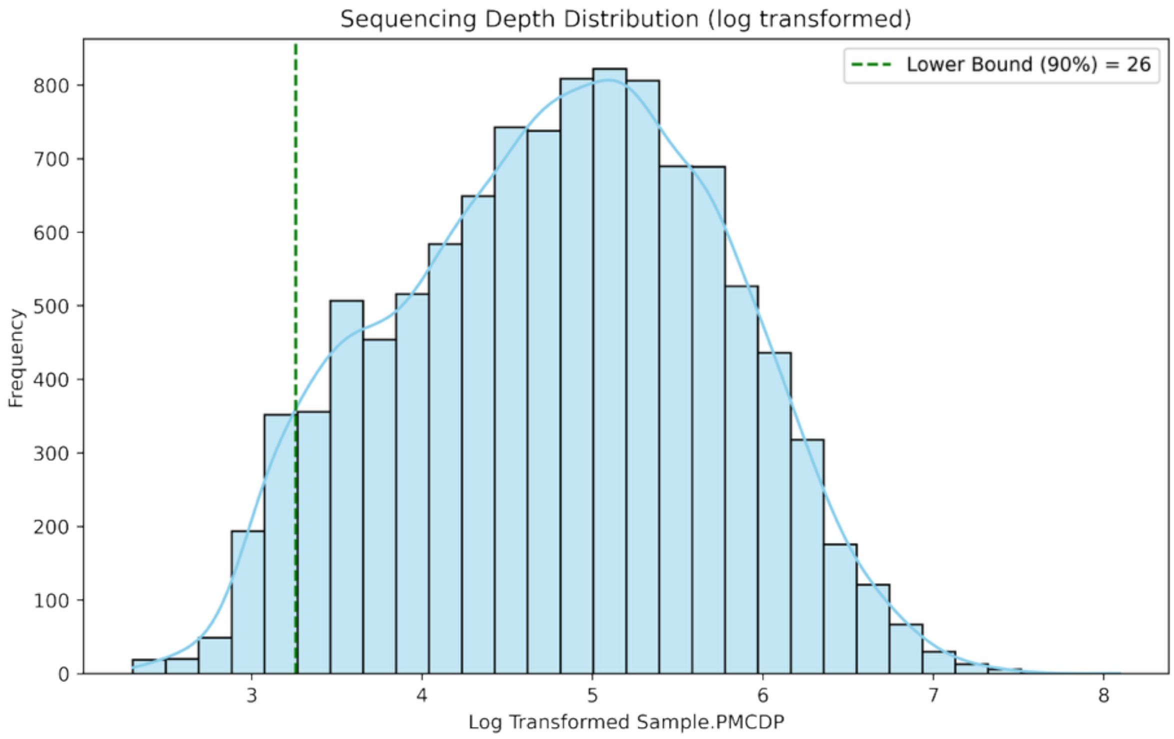

Figure S2. Distribution of sequencing depth (DP) across all samples.

The histogram illustrates the DP distribution, with a kernel density estimate overlaid (blue line). The vertical dashed green line indicates the lower bound threshold for sequencing depth, set at the 10th percentile (DP = 26), ensuring that at least 90% of samples have DP values above this threshold. This empirical cut-off was used to exclude samples with insufficient coverage from downstream analyses.

a.

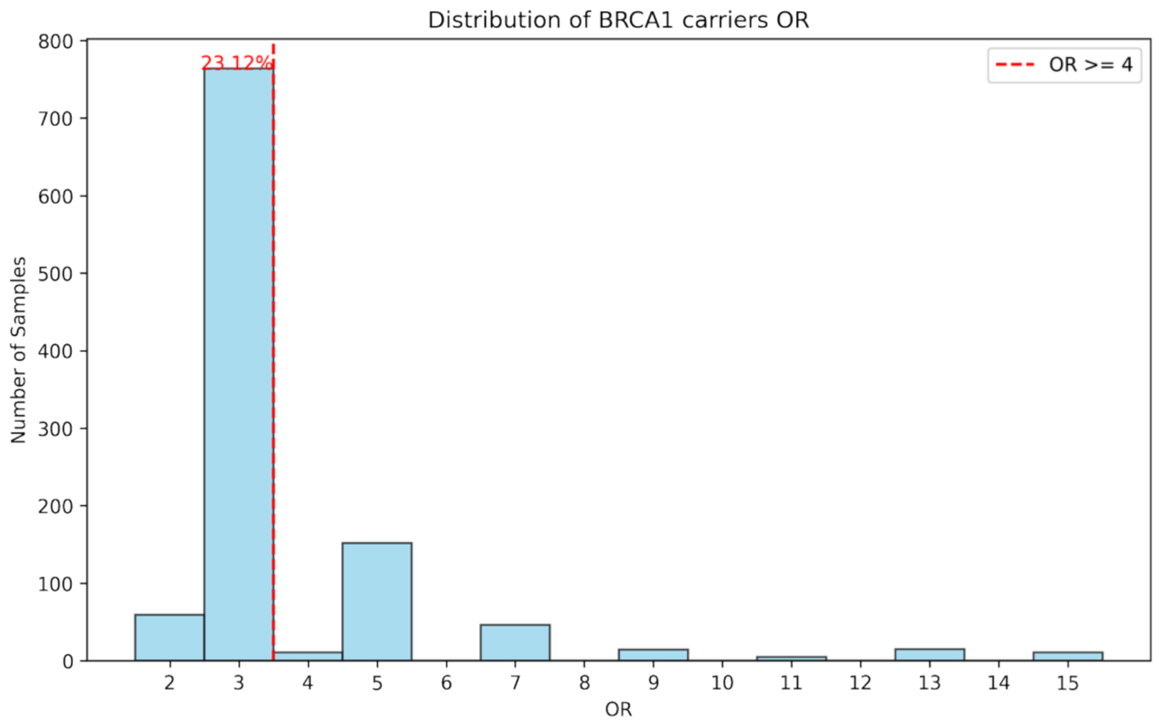

b.

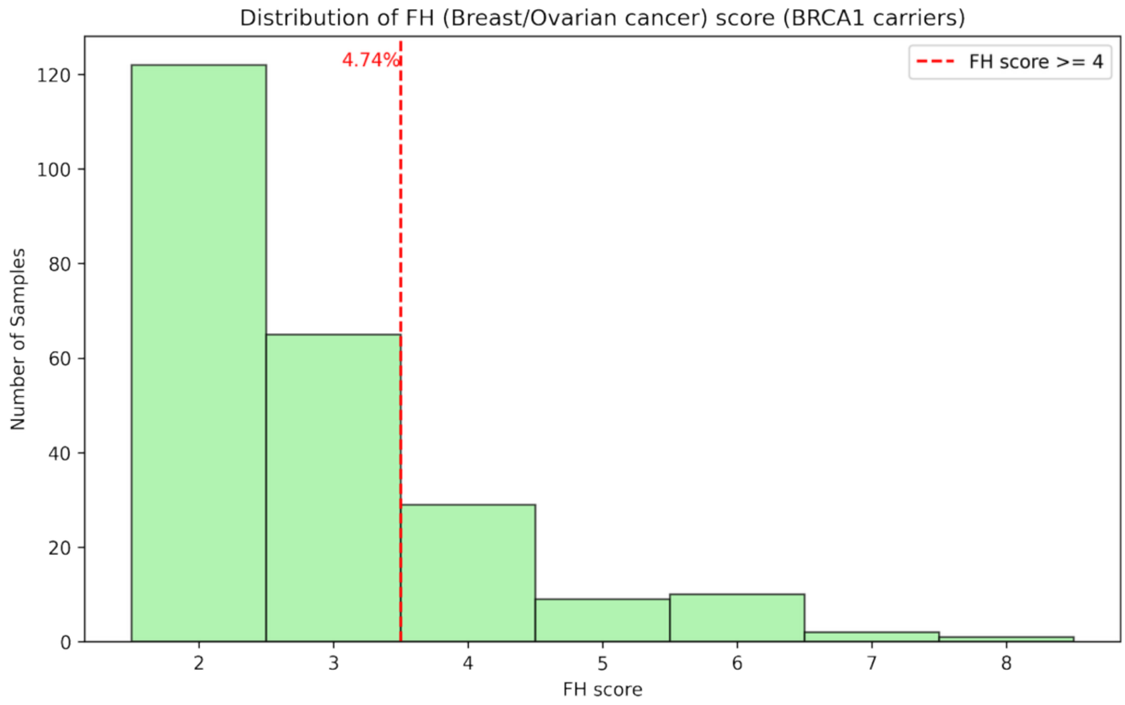

Figure S3. Prioritization of BRCA1 variant for downstream analysis.

(a) Distribution of odds ratios (OR) for all *BRCA1* non-coding variant carriers. The vertical dashed red line marks the threshold for high-priority samples (OR ≥ 4), with the proportion of carriers meeting this criterion (23.1%) indicated above the cutoff.

(b) Distribution of family cancer history scores among *BRCA1* variant carriers. Breast cancer (BC) and ovarian cancer (OC) score was calculated based on the presence of breast and/or ovarian cancer within three generations. The dashed red line indicates the high-priority threshold (FH score ≥ 4), with 4.7% of carriers meeting this criterion.

a.

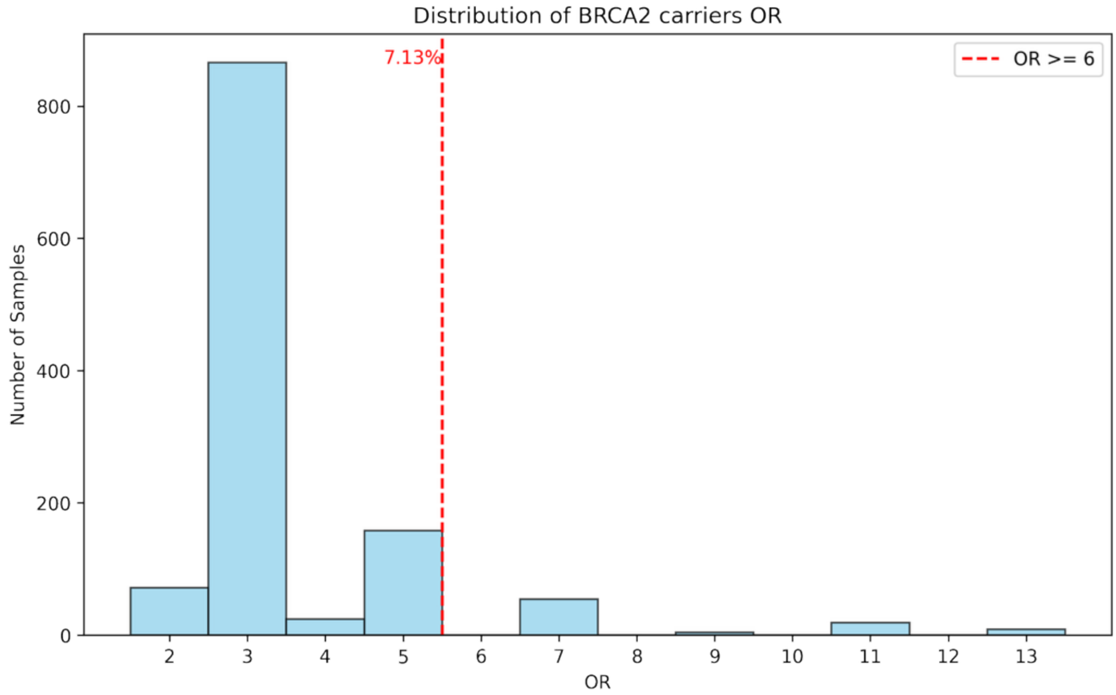

b.

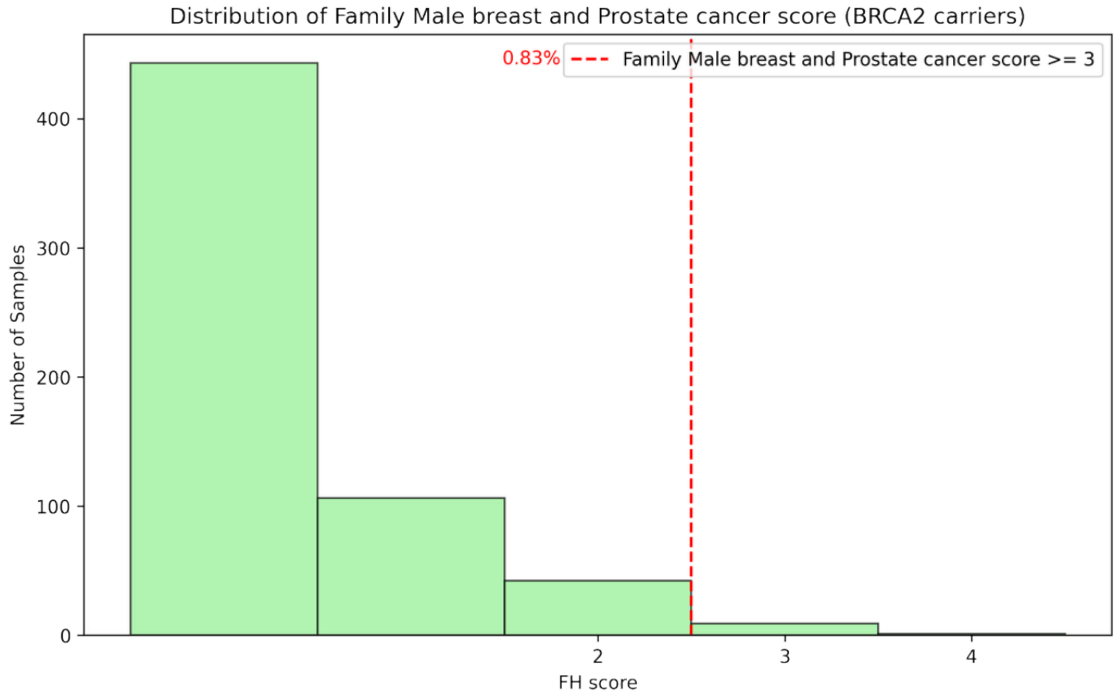

Figure S4. Prioritization of BRCA2 variant for downstream analysis.

(a) Distribution of odds ratios (OR) for all *BRCA2* non-coding variant carriers. The vertical dashed red line denotes the high-priority threshold (OR ≥ 6), with 7.13% of carriers exceeding this value.

(b) Distribution of family cancer history scores among *BRCA2* variant carriers. Male breast cancer (MBC) and prostate cancer (PC) score was calculated as the sum of male breast and prostate cancer cases within three generations. The dashed red line marks the threshold for high-priority samples (MBC and PC score ≥ 3), with 0.83% of carriers meeting this criterion.

a.

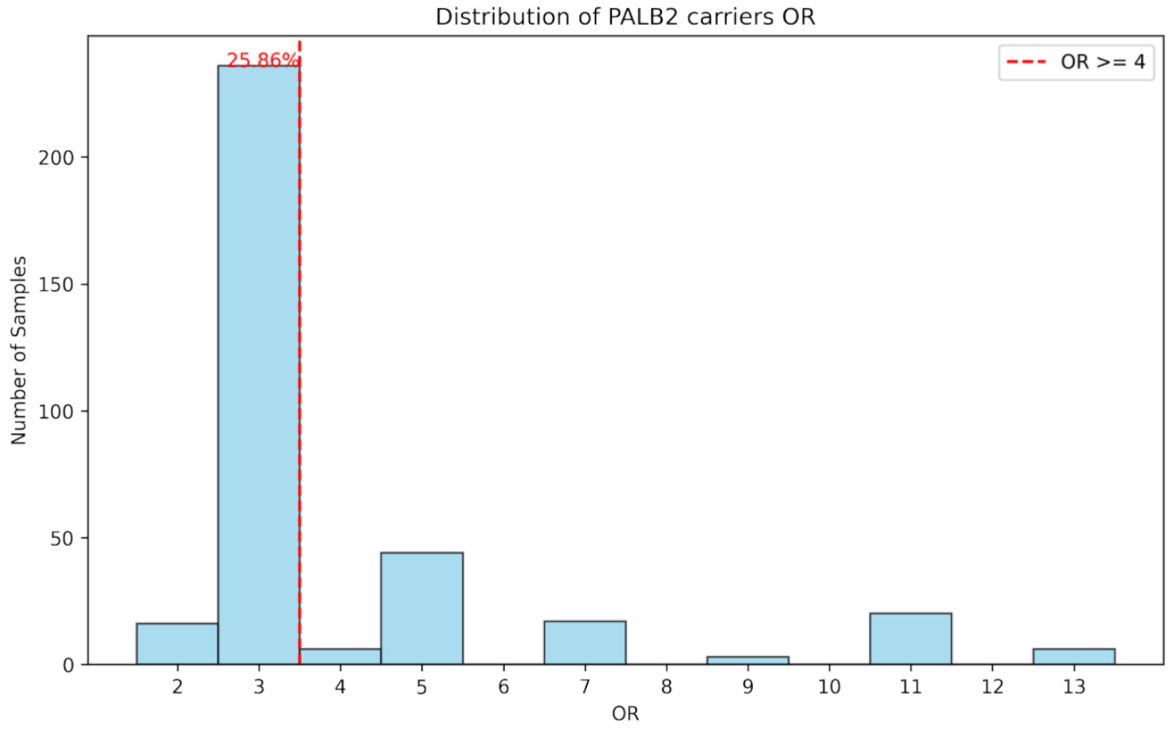

b.

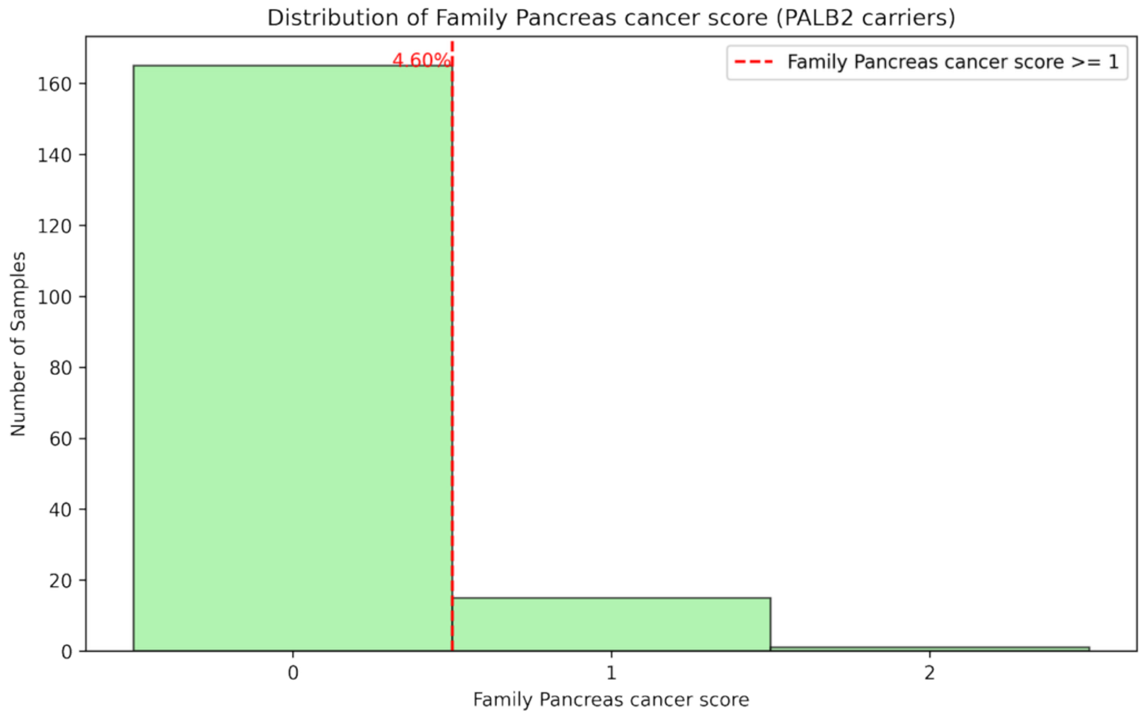

Figure S5. Prioritization of PALB2 variant for downstream analysis.

(a) Distribution of odds ratios (OR) for all *PALB2* non-coding variant carriers. The vertical dashed red line indicates the high-priority threshold (OR ≥ 4), with 25.68% of carriers meeting this criterion.

(b) Distribution of family cancer history scores among *PALB2* variant carriers, calculated based on the number of relatives within three generations affected by pancreatic cancer (PanC). The dashed red line marks the high-priority threshold (PanC score ≥ 1), which was satisfied by 4.6% of carriers.
